# Supplementary material for: Modeling glioblastoma heterogeneity as a dynamic network of cell states
Source: Mol Syst Biol. 2021 Sep 16;17(9):e10105. doi: 10.15252/msb.202010105 (PMC8444284; doi:10.15252/msb.202010105)
Supplement: Supplementary file 6 — Source Data for Figure 5 [file MSB-17-e10105-s004.zip › Figure5A_sourcedata/GSEA_3017/nosigdb_stateA.GseaPreranked.1622795026485/DOROSZKO_U3180MG_CLUSTER_4.html]

Details for gene set DOROSZKO\_U3180MG\_CLUSTER\_4[GSEA]

|  || Dataset | state53017 |
| Phenotype | NoPhenotypeAvailable |
| Upregulated in class | na\_pos |
| GeneSet | DOROSZKO\_U3180MG\_CLUSTER\_4 |
| Enrichment Score (ES) | 0.44594517 |
| Normalized Enrichment Score (NES) | 2.742049 |
| Nominal p-value | 0.0 |
| FDR q-value | 0.0 |
| FWER p-Value | 0.0 |
Table: GSEA Results Summary

  

Fig 1: Enrichment plot: DOROSZKO\_U3180MG\_CLUSTER\_4      
 Profile of the Running ES Score & Positions of GeneSet Members on the Rank Ordered List

  

| PROBE | GENE SYMBOL | GENE\_TITLE | RANK IN GENE LIST | RANK METRIC SCORE | RUNNING ES | CORE ENRICHMENT || 1 | PDGFRA |  |  | 5 | 0.871 | 0.0392 | Yes |
| 2 | KIF11 |  |  | 6 | 0.870 | 0.0837 | Yes |
| 3 | KIF20B |  |  | 10 | 0.749 | 0.1188 | Yes |
| 4 | CEP55 |  |  | 11 | 0.747 | 0.1570 | Yes |
| 5 | PIF1 |  |  | 30 | 0.627 | 0.1699 | Yes |
| 6 | CCNB1 |  |  | 37 | 0.599 | 0.1941 | Yes |
| 7 | HMMR |  |  | 43 | 0.559 | 0.2174 | Yes |
| 8 | GDI2 |  |  | 49 | 0.537 | 0.2395 | Yes |
| 9 | KIF14 |  |  | 59 | 0.503 | 0.2556 | Yes |
| 10 | CENPE |  |  | 62 | 0.496 | 0.2789 | Yes |
| 11 | DLGAP5 |  |  | 76 | 0.475 | 0.2894 | Yes |
| 12 | AURKA |  |  | 78 | 0.474 | 0.3125 | Yes |
| 13 | CDC20 |  |  | 80 | 0.472 | 0.3356 | Yes |
| 14 | PLK1 |  |  | 89 | 0.464 | 0.3508 | Yes |
| 15 | EMP3 |  |  | 101 | 0.452 | 0.3623 | Yes |
| 16 | SEPHS1 |  |  | 114 | 0.437 | 0.3719 | Yes |
| 17 | CDCA8 |  |  | 115 | 0.437 | 0.3942 | Yes |
| 18 | JPT1 |  |  | 125 | 0.426 | 0.4064 | Yes |
| 19 | CCNB2 |  |  | 147 | 0.411 | 0.4051 | Yes |
| 20 | ANLN |  |  | 152 | 0.407 | 0.4216 | Yes |
| 21 | FAM83D |  |  | 180 | 0.389 | 0.4129 | Yes |
| 22 | HNRNPH3 |  |  | 185 | 0.387 | 0.4284 | Yes |
| 23 | BUB1 |  |  | 188 | 0.384 | 0.4459 | Yes |
| 24 | RAB13 |  |  | 220 | 0.366 | 0.4317 | No |
| 25 | CENPA |  |  | 243 | 0.352 | 0.4264 | No |
| 26 | TTK |  |  | 262 | 0.340 | 0.4247 | No |
| 27 | KNSTRN |  |  | 263 | 0.340 | 0.4420 | No |
| 28 | G2E3 |  |  | 302 | 0.325 | 0.4183 | No |
| 29 | SORD |  |  | 317 | 0.320 | 0.4198 | No |
| 30 | TNFAIP8L1 |  |  | 336 | 0.314 | 0.4167 | No |
| 31 | ECT2 |  |  | 365 | 0.305 | 0.4026 | No |
| 32 | UBALD2 |  |  | 387 | 0.299 | 0.3955 | No |
| 33 | DCP2 |  |  | 416 | 0.289 | 0.3806 | No |
| 34 | TMEM160 |  |  | 420 | 0.288 | 0.3921 | No |
| 35 | NCAPD2 |  |  | 440 | 0.283 | 0.3864 | No |
| 36 | EEF1AKMT2 |  |  | 441 | 0.282 | 0.4008 | No |
| 37 | CDC25C |  |  | 443 | 0.282 | 0.4142 | No |
| 38 | KIF20A |  |  | 447 | 0.281 | 0.4254 | No |
| 39 | TXNDC17 |  |  | 468 | 0.276 | 0.4182 | No |
| 40 | UBE2S |  |  | 507 | 0.269 | 0.3917 | No |
| 41 | SPDL1 |  |  | 560 | 0.258 | 0.3496 | No |
| 42 | PLIN3 |  |  | 619 | -0.259 | 0.3013 | No |
| 43 | DCTN3 |  |  | 665 | -0.280 | 0.2678 | No |
| 44 | SDCBP |  |  | 845 | -0.438 | 0.1002 | No |
| 45 | EZR |  |  | 865 | -0.464 | 0.1038 | No |
| 46 | ZNF106 |  |  | 873 | -0.483 | 0.1210 | No |
Table: GSEA details [plain text format]

  

Fig 2: DOROSZKO\_U3180MG\_CLUSTER\_4: Random ES distribution      
 Gene set null distribution of ES for **DOROSZKO\_U3180MG\_CLUSTER\_4**
